# QSAR study of HCV NS5B polymerase inhibitors using the genetic algorithm-multiple linear regression (GA-MLR)

**DOI:** 10.17179/excli2015-731

**Published:** 2016-01-18

**Authors:** Hamid Rafiei, Marziyeh Khanzadeh, Shahla Mozaffari, Mohammad Hassan Bostanifar, Zhila Mohajeri Avval, Reza Aalizadeh, Eslam Pourbasheer

**Affiliations:** 1 Department of Chemistry, Dashtestan Branch, Islamic Azad University, Dashtestan, Iran; 2 Department of Chemistry, Payame Noor University (PNU), P. O. Box 19395-3697, Tehran, Iran; 3 Laboratory of Analytical Chemistry, Department of Chemistry, University of Athens, Panepistimiopolis Zografou, 15771 Athens, Greece

**Keywords:** QSAR, genetic algorithms, multiple linear regression, HCV

## Abstract

Quantitative structure-activity relationship (QSAR) study has been employed for predicting the inhibitory activities of the ***Hepatitis C virus (HCV)*** ***NS5B polymerase inhibitors***. A data set consisted of 72 compounds was selected, and then different types of molecular descriptors were calculated. The whole data set was split into a training set (80 % of the dataset) and a test set (20 % of the dataset) using principle component analysis. The stepwise (SW) and the genetic algorithm (GA) techniques were used as variable selection tools. Multiple linear regression method was then used to linearly correlate the selected descriptors with inhibitory activities. Several validation technique including leave-one-out and leave-group-out cross-validation, Y-randomization method were used to evaluate the internal capability of the derived models. The external prediction ability of the derived models was further analyzed using modified r^2^, concordance correlation coefficient values and Golbraikh and Tropsha acceptable model criteria's. Based on the derived results (GA-MLR), some new insights toward molecular structural requirements for obtaining better inhibitory activity were obtained.

## Introduction

Hepatitis C virus (HCV), identified in 1989 as the etiological agent of parenteral non-A non-B hepatitis, often causes the development of malignant chronic disease, including liver cirrhosis and hepatocellular carcinoma, frequently resulting in death (Alter et al., 1992[3]; Choo et al., 1989[8]; Leyssen et al., 2000[24]). With an estimated 3 % of the global population infected with HCV, including 4.1 million in the United States alone, and no protective vaccine available at present, this disease has emerged as a serious global health problem (Wasley and Alter, 2000[45]; Alter et al., 1999[2]). Although significant advances have been made in the development of treatments for chronic hepatitis C, their efficacy is not universal and only 50 % success has been reported in achieving a sustained viral response for the current combination therapy with new pegylated (PEG) forms of interferon plus ribavirin (Dillon, 2004[11]; Hügle and Cerny, 2003[19]; Walker et al., 2003[43]; Wang and Heinz, 2000[44]). Moreover, this therapy has considerable liabilities including serious adverse side effects and high cost, thus highlighting the need to develop improved therapeutic options to target HCV infections (Cornberg et al., 2003[10]). 

HCV is an envelope positive-stranded RNA virus. Its single-stranded ~9.6 kb RNA genome encodes a large polyprotein of ~3010 amino acids comprising 4 structural proteins (Core, E1, E2, and p7) and 6 nonstructural proteins (NS2, -3, -4A, -4B, -5A, and -5B) (Grakoui et al., 1993[14]; Hijikata et al., 1991[17]; Lohmann et al., 1995[26]). One of the NS proteins, NS5B, an RNA-dependent RNA polymerase (RdRp) is the most studied target for anti-HCV therapy as it is a crucial and unique component of the viral replication machinery (Dillon, 2004[11]; Kaushik-Basu et al., 2007[22]; Wang and Heinz, 2000[44]). NS5B, a 68 kDa membrane-associated protein contains motifs shared by all RdRps in which the catalytic domain is arranged around a central cleft in an organization that resembles a right hand, with the “palm” “finger” and “thumb” subdomains common to polymerases (Bressanelli et al., 2002[7]; Love et al., 2003[27]). Recombinant expression of active, soluble NS5B in a variety of systems has been achieved by various C-terminal deletions between 21 and 55 amino acid residues and its biochemical properties investigated (Kaushik-Basu et al., 2007[22]). All of these reported recombinant HCV RdRps utilize a wide range of RNAs as template *in vitro* without preference, although they do prefer certain homo-polyribonucleotides to others and their activity is stimulated by GTP under specified conditions. Many screening assays for NS5B inhibitors utilize synthetic homopolymeric templates/primers. NS5B inhibitors thus far identified by these screening procedures can be broadly classified as either nucleoside (NI) or non-nucleoside (NNI) inhibitors (Kaushik-Basu et al., 2007[22]). 

Quantitative structure-activity relationships (QSAR) studies play a key role in predicting the biological activity of new compound and provide information that is useful for molecule designing and medicinal chemistry (Karbakhsh and Sabet, 2011[21]; Noorizadeh and Farmany, 2014[31]). QSAR model establishes the mathematical relationship between chemical properties or activities of compounds with their various structural parameters (descriptors) such as topological, physicochemical, stereochemical or electronic indices (Pourbasheer et al., 2014[33]; Rathod, 2011[36]). The most important step in building QSAR models is the selection of one or more molecular descriptors that can represent the true interpretation of molecular structure with its activity or properties (Niazi et al., 2006[30]). Therefore, a validated QSAR model can provide valuable information, not only about the effect of fragments in molecular graph, but also it can predict the biological activities without performing any experimental efforts that the designing results are not clear. In this contribution, multiple linear regression (MLR) technique was employed to build QSAR models using the theoretical molecular descriptors selected by stepwise (SW) and genetic algorithm (GA) methods based on the training set compounds (Li et al., 2008[25]) in order to correlate the biological activities of taken compounds with their chemical strutures. 

The primary goal of this work was to develop a new and validated QSAR model, and then investigating the molecular structural requirements for improving the biological activities based on the derived models.

## Methodology

### Data set

In this study, the data set consisting of 72 molecules of Indole 5-carboxamide derivatives along with their experimental inhibitory activities were taken from the literature (Beaulieu et al., 2011[6][5]). The chemical structures with their activities are shown in Table 1(Tab. 1). The inhibitory activity values [IC_50_ (nM)] were converted to the logarithmic scale pIC_50_ [-log IC_50_ (M)] so as to give numerically larger value, and then used for the subsequent QSAR analyses. The molecules were divided into two subsets using principle component analysis (PCA) in which resulted in generation of the training set contained 59 compounds and the test set contained 13 compounds. The training set was employed to build the model, and the test set was used to evaluate the external prediction ability of the built models. 

### Descriptor calculation

The two-dimensional (2D) structures of the molecules were sketched in Hyperchem v7.3 software (HyperChem, 2002[20]) and pre-optimization was done using molecular mechanics force field (MM+) procedure, and final geometries optimization was performed using semi-empirical (AM1) method with root mean square gradient of 0.01 kcal mol^-1^. A total of 3224 different molecular descriptors were calculated for each molecule using Dragon v5.5 package (Todeschini et al., 2010[41]). The constant or near constant variables were removed, and then, the collinear descriptors (i.e. r>0.9) were removed. The remained molecular descriptors were then taken for variable selection tool to derive the most respective subset of descriptors.

### Principle Component Analysis (PCA)

The division of the dataset into training and test set is the most crucial step since based on the selected compounds, the models are being built. To divide the dataset into training and the test set, principle component analysis (PCA) (Abdi and Williams, 2010[1]) was used so as to split the dataset based on their chemical structures diversity. The compounds in test set were selected considering the distribution in chemical structure diversity and also for avoiding the fitting problem, the better distribution of biological activities for selected compounds were considered. As a result of the PCA, 6 significant principal components (PC-s) were extracted from the variables (PC_1_=49.81 %, PC_2_=22.09 %, PC_3_=12.25 %, PC_4_=7.10 %, PC_5_=6.65 %, PC_6_=3.10 %,). PC_1_ and PC_2_ were selected for the division purpose since they covered the most variability in the dataset. The selection is first made based on the distribution of data points in PC_1_ and PC_2_ and then, the final candidate as test set compounds were chosen by considering the well-distribution for their biological activities. 

### Variable selection technique

The selection of relevant descriptors for building the predictive model is also an important step in model construction. The final goal in this step is to find the most respective descriptors which can be used to predict the biological activities with minimum error. In this contribution, we used two well-known variable selection methods including stepwise (SW) and genetic algorithm (GA). Stepwise regression includes a regression model in which the selecting of predictive variables is done by an automatic procedure (Draper and Smith, 1981[12]) considering the F-test. Stepwise method pursues the forward selection and backward elimination rule where forward selection begins with no variable presented in the model and testing the addition of each variable improving the model outcome while, backward elimination begins with all variable and assessing the removing of variables which can improve the model by being omitted (Draper and Smith, 1981[12]). In genetic algorithms, the initial step is creating a large number of randomly selected descriptors termed chromosome where the variables are included in each chromosome called gene (Holland, 1975[18]; Pourbasheer et al., 2014[32][34]). Despite the stepwise technique, genetic algorithm is not presenting the over fitting issue, since it is using correlation coefficient of leave-one-out cross-validation (Q^2^_LOO_) as a fitness function where subset of variables are being evaluated by their fitness for selection as the most respective descriptors. Subsequently, the subsets with worse fitness function are being excluded and then, the remained subsets are breeding. Finally, the mutation is carrying out. Genetic algorithm technique was first developed by Leardi et al. (1992[23]). Genetic algorithm and stepwise methods as selection tool were written in Matlab 6.5 program (Mathworks, 2005[29]).

## Results and Discussion

The total data set was separated into a training set of 59 compounds to develop the models and a test set of 13 compounds using PCA. The training and test sets are shown in Table 1(Tab. 1). After division of dataset, stepwise method was used to provide the most relevant descriptors for modeling purpose. Multiple linear regression method then was used to linearly correlate the selected descriptors based on the stepwise techniques on the biases of training set compounds, and then evaluated using group of compounds as test set. During the derivation of model, 2 compounds belonging to the test set were detected as outliers and excluded from analyses (Table 1(Tab. 1)). The derived linear equation based on SW-MLR is as follows:

pIC_50_= 22.32 (±3.511) - 4.397 (±0.9607) EEig05x + 2.673 (±0.7931) GGI9 - 0.01958 (±0.008726) RDF065m - 0.7414 (±0.1620) Mor19m + 49.53 (±11.34) R3u+ + 0.1809 (±0.07231) C-028 (1)

*N*_train_= 59, *R*^2^_train_= 0.772, *R*^2^_test_= 0.703, *R*^2^_adj_= 0.745, *F*_train_= 29.284, *F*_test_= 0.9878, *RMSE*_train_= 0.238, *RMSE*_test_ = 0.265, *Q*^2^_LOO_=0.697, *Q*^2^_LGO_= 0.720, *Q*^2^_BOOT_= 0.712, CCC_train_=0.871, CCC_ test_=0.781, r^2^m=0.596, r^2^m _average_=0.433, MAE_train_=0.190, MAE_test_= 0.192.

In above equation, *N* is the number of training set compounds, *R*^2^ is the squared correlation coefficient, *RMSE* is the root mean square error, *R*^2^_adj_ is adjusted *R*^2^, *Q*^2^_LOO_, *Q*^2^_LGO_ and *Q*^2^_BOOT_ are the squared cross-validation coefficients for leave one out, leave group out and bootstrapping respectively, and *F* is the Fisher *F*-statistic. CCC is concordance correlation coefficient and evaluates the degree to which pairs of observations fall on the 45° line through the origin (Pourbasheer et al., 2014[35]). The r^2^m is modified r2 value and MAE is mean absolute error. The developed model since represented lower accuracy for test set, Golbraikh and Tropsha acceptable model criteria's was employed to investigate the reliability of the derived model (Golbraikh and Tropsha, 2002[13]). Four conditions for accepting a model are as follows:

1. Q^2^_LOO_ > 0.5
2. R^2^_test_> 0.6
3. R_0_^2^ - R_0_^'2^/R^2^ < 0.1 and 0.85 < K' < 1.15 or R^2^ - R_0_^2^/R^2^ < 0.1 and 0.85 < K < 1.15
4. R_0_^2^ - R_0_^'2^ < 0.3

where R is correlation coefficient between the observed and predicted values; R_0_^2^ is coefficients of calculation (correlation between predicted versus observed values with intercept of zero), and R_0_′^2^ is correlation between predicted versus observed responses for regressions through the origin; K is slope and K′ is slope of regression lines through the origin. The results of this analysis were listed in Table 2(Tab. 2). As it can be seen, the last condition for acceptance of a derived model based on SW-MLR was rejected. Therefore, the genetic algorithm as a method for variable selection was applied to the same data set (i.e. training and test set selected based on PCA) for selecting the best set of molecular descriptors. The GA-MLR analysis led to a model with six descriptors. This linear model and its statistical parameters are derived as follows:

pIC_50_= 36.97 (±4.056) - 7.971 (±0.9724) EEig05r + 0.6368 (±0.1662) GGI4 - 0.1752 (±0.06418) SPAN - 0.5972 (±0.1320) Mor19m + 45.88 (±13.05) R3u+ - 5.624 (±1.617) R5p (2)

*N*_train_= 59, *R*^2^_train_= 0.792, *R*^2^_test_= 0.713, *R*^2^_adj_= 0.778, *F*_train_= 32.985, *F*_test_=1.3885, *RMSE*_train_= 0.227, *RMSE*_test_ = 0.252, *Q*^2^_LOO_= 0.737, *Q*^2^_LGO_= 0.762, *Q*^2^_BOOT_= 0.731, CCC_train_=0.884, CCC_test_=0.819, r^2^m=0.666, r^2^m _average_=0.533, MAE_train_=0.188, MAE_test_= 0.213.

**Figure 1:**
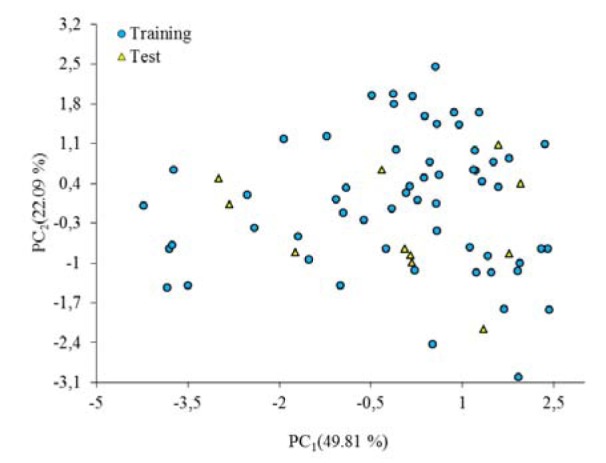
Principle component analysis with PC_1_ and PC_2_ with test set for GA-MLR result

**Figure 2:**
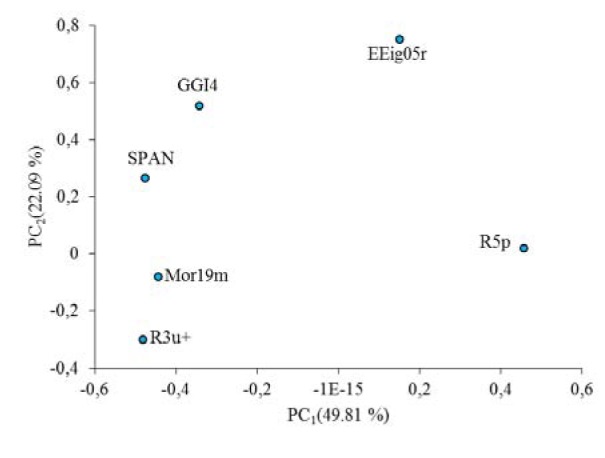
PC_1_-PC_2_ loadings plot using the six descriptors for the best model (GA-MLR)

The PCA results were shown in Figure 1(Fig. 1). PC_1_-PC_2_ loadings plot using the six descriptors for the best model (GA-MLR) were shown in Figure 2(Fig. 2). In Figure 2(Fig. 2), for the loadings it is confirmed that the compounds with higher biological activity values, located on the left side which are presenting a large contribution of the R3u+ descriptor, situated on the same side in Figure 1(Fig. 1). On the other hand, compounds with lower biological activity values, on the right side, have more pronounced contributions from the other descriptors (mostly from R5p and EEig05r). Also it can be observed that the distribution of scores in Figure 1(Fig. 1) is much more in right side and upper which represent that the most of compounds in data set have higher value for descriptors that have negative values than for the descriptors with positive effects. Therefore, the selected PCs are the true representative of the molecular descriptors that can be encoded for understanding the correlation between chemical structures and biological activities.

**Figure 3:**
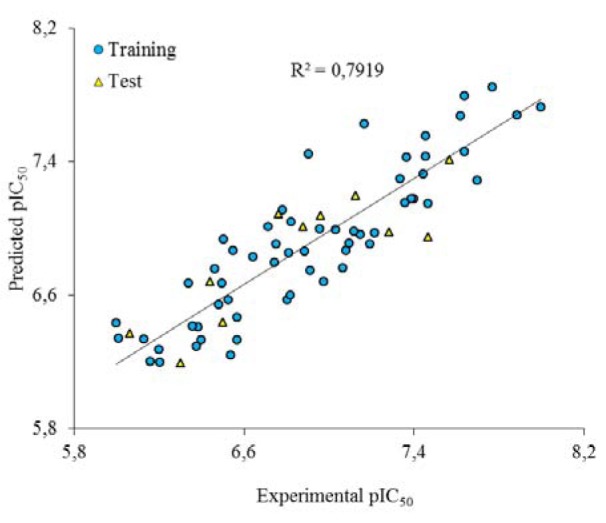
The predicted pIC_50_ values by the GA-MLR modeling vs. the experimental pIC_50_ values

Golbraikh and Tropsha acceptable model criteria's was employed for evaluating the prediction capability of the built GA-MLR model. The results are listed in Table 2(Tab. 2). As it can be seen, the all conditions were accepted for GA-MLR and therefore, it was used as a main model for prediction purpose. The experimental and predicted activities based on this model were given in Table 1(Tab. 1). The plot of the predicted pIC_50_ versus the experimental pIC_50_ is demonstrated in Figure 3(Fig. 3). As can be seen from Table 1(Fig. 1) and Figure 3(Fig. 3), the calculated activity values are in good agreement with experimental activity values.

The inter-correlation between the six selected descriptors was inspected by calculating their variance inflation factor (VIF), which are also given in Table 3(Tab. 3). The VIF values, calculated as 1/1- r^2^, where r^2^ is the multiple correlation coefficient of one descriptor's effect regressed on the remaining molecular descriptors. If VIF equals to 1, then no inter-correlation exists for each variable; if VIF falls into the range between 1 and 5, the related model is acceptable; and if VIF is larger than 10, the related model is unstable and a recheck is necessary (Maryam et al., 2012[28]). As it can be seen by the given information of Table 3(Tab. 3), most of the variables had VIF values of less than 5, indicating that the GA-MLR model has statistic significance. 

The built GA-MLR model was validated using the leave-one-out and leave-group-out cross-validated correlation coefficients (Q^2^_LOO_ and Q^2^_LGO_). The robustness of the GA-MLR model and its predictive ability was confirmed by the high Q^2^_BOOT_ source based on bootstrapping repeated 5000 times (Hadizadeh et al., 2013[15]). The results produced by the Q^2^_LOO_, Q^2^_LGO_ and Q^2^_BOOT_ parameters along with other validation parameters showed the higher quality of the developed GA-MLR model. Therefore, this model can be used to predict the inhibition activity of the compounds. 

**Figure 4:**
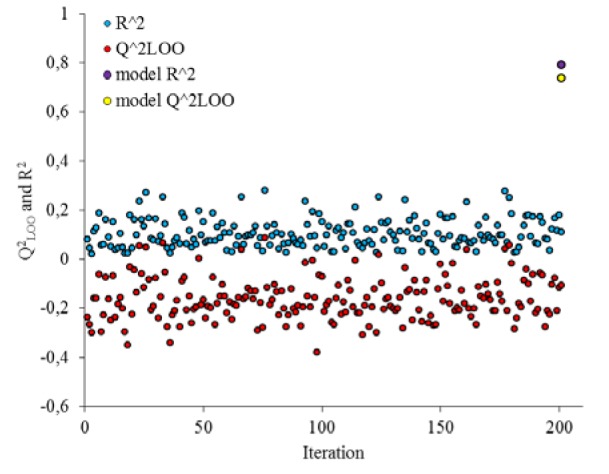
*R*^2^_train_ and *Q*^2^_LOO_ values after several Y-randomization tests for GA-MLR

The robustness of the QSAR model was further assessed by applying Y-randomization test. The dependent variable vector (inhibitory activity) was shuffled randomly and the new QSAR models (after several repetitions) would be anticipated to have low R^2^ and Q^2^_LOO_ values (Figure 4(Fig. 4)) (Asadollahi et al., 2011[4]). As it can be seen from Figure 4(Fig. 4), after 200 times shuffling the biological response for compounds, all of the derived new models were less than that of obtained in real response.

**Figure 5:**
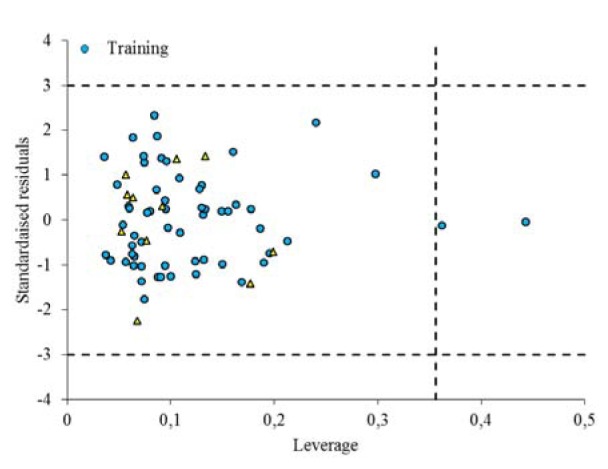
The William plot for the predictive GA-MLR model

The Williams plot, the plot of the standardized residuals versus the leverage (h), is used to visualize the applicability domain (AD) of QSAR models (Vahdani and Bayat, 2011[42]). From the Williams plot (Figure 5(Fig. 5)), it is obvious that there are only two compounds (No. 1 and No. 6 belonging to the training set) have the leverage higher than the warning *h*∗ value of 0.356, thus they can be considered as structural outliers. From Figure 4(Fig. 4), it is obvious that the standardized residuals observed for all the compounds in the training and test sets are smaller than three standard deviation units (3δ). Thus, the generated model is acceptable for prediction purpose.

### Interpretation of descriptors

By interpreting the descriptors contained in GA-MLR model, some new insights can be obtained which can be helpful for understanding the correlation of chemical structure with biological activities.

The first selected descriptor is Eigenvalue 05 from edge adj. matrix weighted by resonance integrals (EEig05r) which belongs to the edge adjacency indices and encodes the connectivity between graph edges (Todeschini and Consonni, 2000[39]). Resonance is a kind of energy stabilizing because of its delocalization effects over electrons in a bond network. As it can be seen, this descriptor represented negative effect in derived GA-MLR model encoding that increasing in the value of EEig05r by increasing the capability of the molecules (the functional groups that provide resonance in bonding with other part of bonding network) for providing more resonances would cause to decrease the pIC_50 _of compounds.

GGI4 is the second selected descriptor which is representing the topological charge index of order 4 (Todeschini and Consonni, 2008[38]). Topological charge indices are evaluating the charge transfer between atoms. These types of descriptors were first introduced by Galvez. In this concept a matrix called **M** was being obtained by multiplying the adjacency matrix **A** by the reciprocal square distance matrix (**D^-2^**). However to prevent the division by zero, the diagonal entries of the distance matrix remain the same; the obtained matrix **M** called the Galvez matrix is then the unsymmetrical matrix (*A×A*) , and A is the number of atoms in matrix. Based on the derived **M** matrix the charge term matrix (**CT_ij_**) which is the charge transfer between the pair of considered vertices can be obtained as follows:


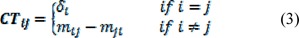


where *m_ij_* is elements of matrix M, *δ_i_* is vertex degree of *i* atom. *CT_ij_* is also representing the net charge transfer between atom *j* and *i*. Hence, for each path length *k*, a topological charge index termed as *G_k_* can be obtained as follows:


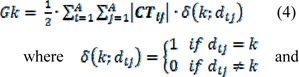


*d_ij_* is elements of distance matrix. Therefore, the *G_k_* is the half-sum of all charge and indicate the total charge transfer between atoms placed at topological distance k. The positive sign of this descriptor in derived linear equation indicates that increasing the charge transfer between the pair of atoms would result in increase of the pIC_50_ values, respectively. 

The third selected descriptor (SPAN) is span R which belonged to geometrical size indices and represents the radius of the smallest sphere, centered on the mass, enclosing all atoms of a molecule (Todeschini and Consonni, 2009[40]), and can be calculated as follows:


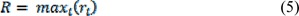


where *r_i_* is the distance of the *ith* atom from the center of the mass. Since this descriptor represents the negative sign in derived linear model, increasing the size of molecules by increasing the distance of specific moieties in molecules would result in decrease of the pIC_50 _values.

Mor19m, the fourth selected descriptor of GA-MLR equation, 3D-MoRSE-signal 19/weighted by atomic masses, belongs to the 3D-MoRSE descriptors. This group of descriptors is subgroup of geometrical descriptors (Todeschini and Consonni, 2000[39]). Value of this group of descriptors is dependent to 3D structure of molecule. 3D-MoRSE descriptors (3D-Molecule Representation of Structures based on electron diffraction) are based on the idea of obtaining information from the 3D atomic coordinates by the transform used in electron diffraction studies for preparing theoretical scattering curves (Soltzberg and Wilkins, 1977[37]). This can be performed by infrared spectra simulation using a generalized scattering function. The Mor19m is associated with negative regression coefficient indicating that decreases in the corresponding 3D-MoRSE signal at scanning distance of 19 would result in increase of pIC_50_value, namely.

The fifth and six descriptors (R3u+ and R5p, respectively) belong to the GETAWAY R-indices descriptors. GETAWAY descriptors are for geometry, topology and atomic-weights assembly. These descriptors are geometrical descriptors in which provide good position of substituents and fragments in molecule (Consonni et al., 2002[9]). In addition, they can carry on good information on molecular size and shape. R3u+ (R maximal autocorrelation of lag 3/unweighted) related to the maximum steric contributions to molecules shape with the topological distance of 3 (Hall and Kier, 1995[16]; Todeschini and Consonni, 2000[39]). Since it presented a positive sign in derived linear equation, increasing in value of this descriptor will cause to increase of the activity (pIC_50_). On the other hand, the other type of GETAWAY R-indices (i.e. R5p) which is R maximal autocorrelation of lag 5/weighted by polarizability would cause decrease in biological activity (pIC_50_) due to its negative sign in obtained linear equation. Therefore, to obtain a good biological activity, the polarizibility of molecule should be decreased.

To conclude, it was observed that the capability of having more resonances in molecular graph is not appropriate and since most of the functional groups belonging to polar groups can represent the presence, therefore, the replacing of more polar groups should be avoided addressing to the negative effect of EEig05r and R5p descriptors. It was also seen that distance of substituents from mass center would cause negative effect on biological activities. However, a good biological activity can be presented if the charge transfer between bonding network and steric contributions to molecules shape increase.

## Conclusion

A robust QSAR model was developed based on PCA-GA-MLR for a dataset consisting of 72 HCV NS5B polymerase inhibitors. The derived models were validated based on several validation techniques, and it was observed that GA-MLR is more accurate than the derived SW-MLR model. Based on the obtained results of GA-MLR, it was observed that the capability of having more resonances in molecular graph is not appropriate and since most of the functional groups belonging to polar groups can represent the presence, therefore, the replacing of more polar groups should be avoided addressing to the negative effect of EEig05r and R5p descriptors. It was also seen that distance of substituents from mass center would cause negative impact over biological activities. However, a good biological activity can be presented if the charge transfer between bonding network and steric contributions to molecules shape increase. In this work, the proposed models could identify and provide better insights about the chemical structure requirements for increasing the pIC_50_ values.

## Figures and Tables

**Table 1:**
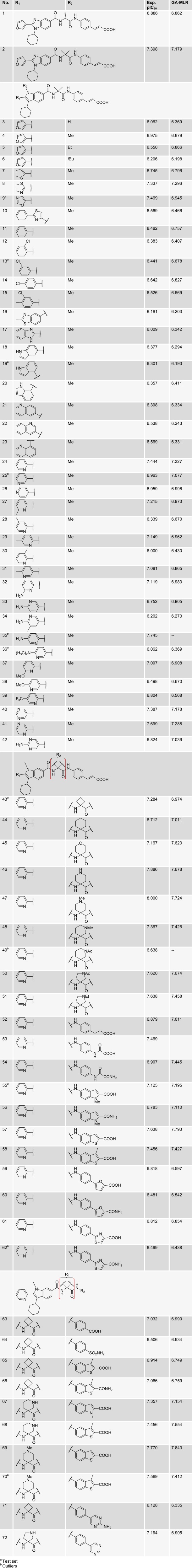
Table1: Chemical structures and the corresponding observed and predicted pIC_50_ values by GA-MLR method

**Table 2:**
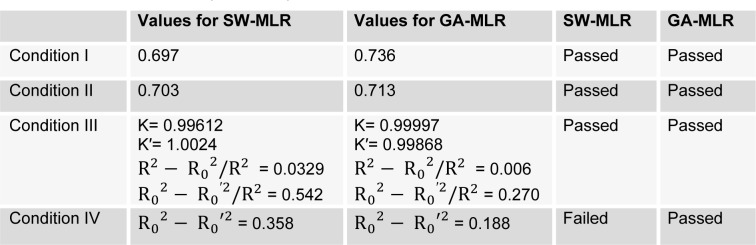
Golbraikh and Tropsha acceptable model criteria's for SW-MLR and GA-MLR

**Table 3:**
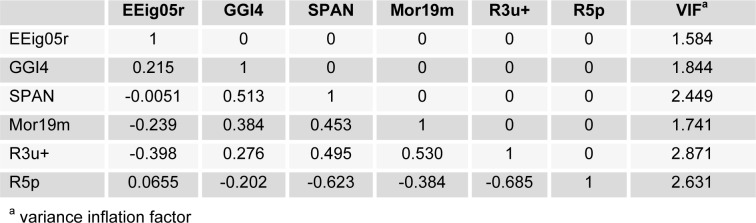
Correlation coefficient matrix of the selected descriptors with their VIF values
